# Hepatic progenitor cell activation and ductular reaction in metabolic dysfunction-associated steatotic liver disease (MASLD): Indicators for disease activity and the degree of fibrosis: The pilot study

**DOI:** 10.1097/MD.0000000000042108

**Published:** 2025-04-11

**Authors:** Melek Büyük, Neslihan Berker, Sidar Bağbudar, Bilger Çavuş, Mine Güllüoğlu

**Affiliations:** a Department of Pathology, Istanbul University, Istanbul Faculty of Medicine, Istanbul, Turkey; b Department of Gastroenterology and Hepatology, Istanbul University, Istanbul Faculty of Medicine, Istanbul, Turkey.

**Keywords:** ductular reaction, hepatic progenitor cell activation, immunohistochemistry, metabolic dysfunction-associated steatotic liver disease, pathologic findings, steatohepatitis

## Abstract

Metabolic dysfunction-associated steatotic liver disease (MASLD) spectrum encompasses steatosis, metabolic dysfunction-associated steatohepatitis, fibrosis, cirrhosis and metabolic dysfunction-associated steatohepatitis-related hepatocellular carcinoma. We evaluated the histomorphologic findings, portal-periportal biliary epithelial cell changes, and factors that may be associated with the degree of fibrosis in liver biopsies of MASLD patients. Hematoxylin-eosin, masson-trichrome, keratin7, keratin19, CD34, and glutamine synthetase-stained biopsies of 34 patients and 10 healthy liver donors (as controls) were retrospectively analyzed. Lobular inflammation was significantly correlated to the ballooning degeneration (*P* = .023), portal inflammation (*P* = .003), ductular reaction (DR) grade (*P* = .027), and the degree of fibrosis (*P* = .003). Ballooning degeneration (*P* = .004), and NAS (*P* = .008) were significantly related to the degree of fibrosis. Portal inflammation had a significant relationship with both DR grade (*P* < .001) and the degree of fibrosis (*P* = .002). The presence of hepatic progenitor cells (HPCs) was related to NAS (*P* = .005) and correlated with the DR grade (*P* = .002) and the degree of fibrosis (*P* = .038). Both DR (*P* < .001) and biliary metaplasia (*P* = .024) were significantly correlated with the degree of fibrosis. In multivariate analysis, biliary metaplasia (*P* = .015) and DR (*P* = .02) were found to be independent factors related to degree of fibrosis. Our results showed that HPC and DR were closely associated with disease activity and degree of fibrosis and might be good indicators of disease progression in MASLD. As pathologists, we might integrate the degree of HPCs and the grade of DR in our pathology reports as these findings might contribute to the disease progression risk categorization of the patients.

## Graphical abstract

**Figure:**
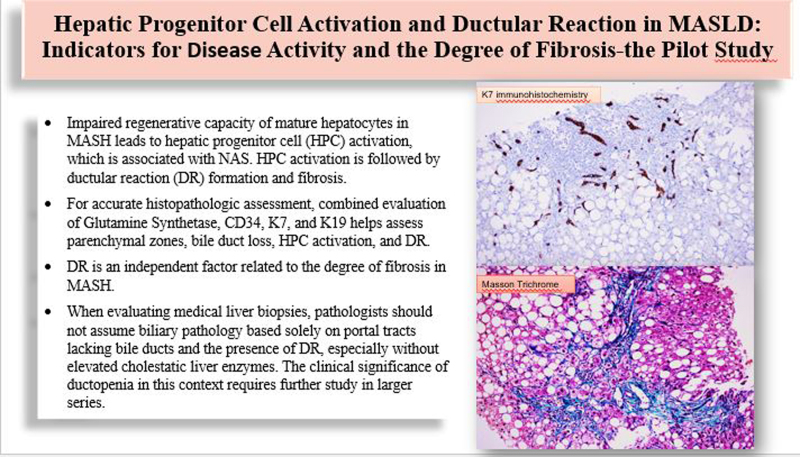

**Key points**

- Impaired regenerative capacity of mature hepatocytes in MASH leads to hepatic progenitor cell (HPC) activation, which is associated with NAS. HPC activation is followed by ductular reaction (DR) formation and fibrosis.
- For accurate histopathologic assessment, combined evaluation of glutamine synthetase, CD34, K7, and K19 helps assess parenchymal zones, bile duct loss, HPC activation, and DR.
- DR is an independent factor related to the degree of fibrosis in MASH.
- When evaluating medical liver biopsies, pathologists should not assume biliary pathology based solely on portal tracts lacking bile ducts and the presence of DR, especially without elevated cholestatic liver enzymes. The clinical significance of ductopenia in this context requires further study in larger series.

## 1. Introduction

Metabolic dysfunction-associated steatotic liver disease (MASLD), formerly known as nonalcoholic fatty liver disease, has become the most common chronic liver disease and a major leading cause of cirrhosis worldwide due to the increasing prevalence of obesity and diabetes mellitus.^[1,2]^ The global prevalence of MASLD is estimated to be 25%.^[3]^ Many studies have generated predictions about the frequency of MASLD-related cirrhosis in various countries by 2030 and the smallest projected increase in the prevalence of decompensated MASLD-associated cirrhosis cases was 65% and the largest projected increase was 187%.^[4]^

MASLD spectrum encompasses steatosis, metabolic dysfunction-associated steatohepatitis (MASH) (formerly known as nonalcoholic steatohepatitis), fibrosis, cirrhosis and MASH-related hepatocellular carcinoma.^[2,5,6]^ MASH is characterized by the presence of hepatocellular injury, observed as ballooning degeneration in addition to steatosis. Unlike simple steatosis, MASH has the potential to progress into advanced liver disease, that is, cirrhosis, and hepatocellular carcinoma.^[1,7]^ Because of its growing incidence and capability of progression to cirrhosis, MASH has been considered the second most common cause of indication for liver transplantation.^[8]^

Although radiologic imaging techniques such as ultrasonography, magnetic resonance imaging, and computed tomography can reveal the degree of liver steatosis, the definitive diagnosis of MASH and the exclusion of other etiologies, including those in the differential diagnosis, require liver biopsy. The level of inflammatory activity, the severity of fibrosis, the risk of progressive disease and prognosis can be accurately assessed through liver biopsy.^[9,10]^ Due to the rising incidence of MASLD in the population and its potential progression to cirrhosis, liver biopsy plays a significant role in clinical management.

Research on factors influencing disease progression in MASLD has suggested that fibrosis may develop in individuals with MASH, likely driven by necro-inflammation.^[11,12]^ Steatonecrosis at the pericentral area often leads to pericellular/perisinusoidal fibrosis in zone 3, which is a common fibrosis pattern in MASH.^[13]^ The distinctive perisinusoidal fibrosis pattern can progress without developing periportal fibrosis, but it usually does develop.^[14]^ However, as fibrosis progresses in portal/periportal areas and bridging develops, it can advance to cirrhosis.

In MASLD, the oxidative liver damage leads inhibition of mature hepatocyte replication, and this results in activation of bipotential cells which are known as progenitor cells that can activate and differentiate into hepatocytes or biliary epithelial cells during liver injury.^[15–17]^ Hepatic progenitor cells (HPCs) were revealed as small-sized isolated oval cells or cell strains with epithelial characteristics, without discernable lumen in periportal and porto-septal areas.^[16–19]^ It was concluded that HPC activation and DR are associated with portal/periportal fibrosis and disease progression.^[16,18,19]^ The term DR which has been described by Roskams et al is a response to inflammation and characterized by ductular proliferation or hyperplasia. DR involves not only ductular proliferation but also reactions involving other cells such as stromal cells, inflammatory cells, and bone marrow-derived macrophages.^[20]^ It is known that HPC differentiation is the core element of DR.^[15]^

In this study, we aimed to assess the histologic findings in liver biopsies of MASLD patients and their association with the degree of fibrosis. Additionally, we aimed to identify portal-periportal biliary cell changes, including interlobular bile ducts appearance, HPC activation, and DR, using cytokeratin 7 (K7) and cytokeratin 19 (K19) immunohistochemistry, and to elucidate their relationship with the degree of fibrosis and liver enzyme levels.

## 2. Materials and methods

### 2.1. Patients

We retrospectively analyzed our pathology records between 2018 and 2023 for liver core biopsies of patients with clinical diagnosis of MASLD. Patients with histologic features of fatty liver disease combined with other liver diseases (such as primary sclerosing cholangitis, primary biliary cholangitis, Wilson disease, autoimmune hepatitis, chronic viral hepatitis, and drug-induced liver injury) or those with clinical information suggesting a liver disease other than MASLD, even without histologic findings, were excluded from the study. Out of the patients initially reviewed, 34 met our criteria and were included in the study. Clinical and laboratory data were retrieved from clinical records.

Liver biopsies of 10 living-related liver transplant donors were also included in the study as control group. The liver biopsies of both study and control group cases had been obtained percutaneously under ultrasound guidance.

This study was approved by the Clinical Research Ethics Committee of the xxx University Faculty of Medicine (file number: 2024/1081). This is a retrospective study; therefore, informed consent was not included.

### 2.2. Histologic evaluation

All the archived Hematoxylin-Eosin (H&E) and Masson-Trichrome (MT) stained liver biopsy slides were reexamined, and histologic findings were documented by 2 experienced liver pathologists (MG, MB). The diagnosis of MASH was defined as the presence of steatosis accompanied by hepatocyte ballooning degeneration and inflammation with or without fibrosis.

Histologic disease activity, determined by the degree of lobular inflammation, ballooning degeneration and steatosis were assessed on H&E-stained sections and graded according to the widely used NAFLD activity score (NAS).^[21]^ The severity of steatosis graded as 0 to 3 based on the percentage of hepatocytes in the biopsy involved (0: <5%; 1: 5–33%; 2: 34–66%; 3: >66%). Hepatocytes with a rounded shape and pale cytoplasm, either similar in size to normal hepatocytes or enlarged up to twice the size of normal hepatocytes, were indicative of ballooning degeneration.^[22]^ The severity of hepatocellular ballooning degeneration, based on the numbers of hepatocytes showing this change, was scored as 0 to 2 (0: none; 1: few ballooned cells; 2: many ballooned cells). The degree of lobular inflammation evaluated and scored 0– to 3 (0: no foci; 1:2 foci per 200× field; 2: 2–4 foci per 200× field; 3: >4 foci per 200× field). NAS ranged from 0 to 8, including scores for steatosis (0–3), lobular inflammation (0–3), and hepatocellular ballooning (0–2).^[21]^

The degree of portal inflammation was graded according to the widely used method by Ishak et al and a score of 0 to 4 was assigned for portal inflammation.^[23]^

The degree of fibrosis was assessed on MT-stained sections (0: none; 1A: mild, zone 3, perisinusoidal; 1B: moderate, zone 3, perisinusoidal; 1C: portal-periportal; 2: perisinusoidal and portal/periportal; 3: bridging fibrosis; 4: cirrhosis).^[21]^

### 2.3. Immunohistochemistry

We utilized K7 and K19 immunohistochemistry to evaluate the interlobular bile ducts and determine the presence of HPC activation, DR, and biliary metaplasia. We performed glutamine synthetase (GS) immunohistochemistry to identify portal tracts lacking bile ducts, relying on its staining pattern in zone 3 hepatocytes around the hepatic vein as a guide. Additionally, we also utilized CD34 immunohistochemistry to reveal the central venule and portal/periportal vascular structures.

Four µm-thick tissue sections of the biopsies of MASLD and control cases were incubated with the primary antibodies K7 (OV-TL 12/30, cell marque, 2 hour incubation), K19 (A53-B/A2.26, Scytek, 32 minute incubation), GS (6/GS, BioCare, 1 hour incubation), and CD34 (qbend/10, Novocastra, 1 hour incubation) by using an automated staining module (Ventana Medical System-Benchmark XT/ISH Staining Module, Roche, Switzerland).

### 2.4. Immunohistochemical evaluation

The number of HPCs in each biopsy was assessed by calculating the average count per field using a 20× objective, based on cell counts in 3 to 5 nonoverlapping fields.^[24]^ Only single cells or cell strains lacking a discernible luminal architecture were considered as HPCs. Reactive ductules without a distinct lumen in the edges of portal areas were accepted as DR and graded based on a previous study.^[25]^ Category “0” indicates no or minimal DR around a few portal tracts and septa; “+/mild” indicates focal DR around most portal tracts/septa; “++/moderate” indicates continuous DR around <50% of portal tracts/septa; and “+++/severe” indicates continuous DR around more than 50% of portal tracts/septa.

Hepatocytes with characteristic morphologic features and cytoplasmic K7 immunoexpression (i.e., intermediate hepatocytes) were accepted as biliary metaplasia.

Normal GS expression was defined as a rim of 2 to 3 layers of strongly positive hepatocytes around the hepatic veins (perivenular pattern).^[26]^ Staining patterns different from this were considered unusual.

Portal tracts lacking interlobular bile ducts were determined by combined evaluation of GS, CD34, K7, K19 slides and then the portal tracts lacking bile ducts were counted. The cases were grouped according to the percentage of portal tracts lacking bile ducts as <10%, 10% to 49%, 50% to 75%, and >75%. In case of exceeding 50% of the portal tracts lacking bile duct was accepted as ductopenia.

### 2.5. Statistical analysis

Continuous normally distributed variables were remarked as the mean ± the standard deviation (SD) of the mean. The Student *t* test and Mann–Whitney U tests were used to compare medians in normally and non-normally distributed data sets, respectively. The Pearson correlation coefficient was used to calculate correlations between continuous normally distributed variables. The Spearman nonparametric correlation was used to determine the strength of the relationship between nonparametric or ordinal variables.

Multivariate logistic or linear regression analysis was used for multivariate analysis to identify predictors for stage of fibrosis and the associated risk in terms of odds ratio (OR) and 95% confidence intervals (CI)). For this analysis, a backward review was performed to obtain better quantification within groups. Due to the low number of cases in some groups, stages 3 and 4 were grouped together for fibrosis staging, while scores 0 and 1 were combined for lobular inflammation, and scores 3 and 4 were grouped for portal inflammation. Additionally, cases with NAS < 3 and 3 to 4 were combined into a single group.

All statistical analyses were performed using the Statistical Package for Social Sciences (SPSS) software for Windows version 25.0 (IBM Corp, Armonk, NY). A *P* value of <.05 was accepted statistically significant.

## 3. Results

### 3.1. Patient characteristics

The study cohort included 34 patients of which 15 were males (44.1%) and 19 females (55.8%). The mean age was 43 years (±15.3 SD; range, 14–72). Male patients were significantly younger (mean age was 33.3 [±14.2 SD]) than the female patients (mean age was 50.8 (±11.5 SD) (*P* < .001).

In the control group, the mean age was 41 (±12 SD; range, 24–62) years and included 5 males (50%) and 5 females (50%).

The biopsy indication was elevated liver aminotransferase levels in 28 patients. The liver biopsy was taken for the assessment of steatosis for the remaining 6 patients with normal liver enzymes.

Serum aspartate aminotransferase (AST), alanine aminotransferase (ALT), gamma-glutamyl transferase (GGT), and alkaline phosphatase (ALP) elevations were observed in 20 (59%), 24 (70%), 13 (38%), and 12 (35%) patients, respectively, at the time of the biopsy. The mean values of AST, ALT, GGT, and ALP were 55 IU/L (range, 19–139), 91 IU/L (18–330), 109 IU/L (16–841), and 105 IU/L (43–256), respectively. Hyperbilirubinemia was observed in 5 of the 31 patients (16%).

### 3.2. Histologic findings

The mean length of the liver biopsies in the study group was 2 cm (range 0.4–4 cm), while in the control group, it was 2.3 cm (range 1.2–3 cm). The mean number of portal tracts in the study and control groups was 24 (range 9–47) and 21 (range 11–34), respectively.

Grade 1 steatosis was detected in 14 (41%) cases, grade 2 in 11 (32%), and grade 3 in 9 (27%) cases. In 5 cases (14.7%) score 1, in 19 cases (56%) score 2, in 9 cases (26%) score 3 lobular inflammation were observed. Lobular inflammation was not detected (score 0) in one (3%) case. Twenty cases (59%) presented a few ballooned cells, 11 cases (32%) had many ballooned cells, while ballooning degeneration was absent in 3 cases (9%). NAS was < 3 in 2 cases (6%), 3 to 4 in 11 (32%), and 5 to 8 in 21 (62%) cases.

Grade 1 portal inflammation was detected in 11 cases (32%), while grade 2 was observed in an equal number (32%) of cases. Grade 3 portal inflammation was identified in 3 (9%) cases, and grade 4 in 1 (3%) case. Additionally, portal inflammation was notably absent in 8 (24%) cases.

In the evaluation of MT-stained slides, 4 (12%) of the MASLD cases showed no fibrosis, fourteen cases (41%) displayed stage 1 fibrosis, eleven cases (32%) displayed stage 2 fibrosis, 4 cases (12%) displayed stage 3 fibrosis, and one case (3%) had cirrhosis.

There were no inflammation, steatosis, or fibrosis in the control group.

Demographic, histologic, and laboratory findings are summarized in Table 1.

**Table 1:** Demographic, histologic and laboratory findings in MASLD patients.

	n: 34 (%)
**Age (years, mean ± SD**)	43 (±15,3 SD)
**Gender**	
Female	19 (55.8%)
Male	15 (44.1%)
**Steatosis**	
Grade 0	0 (0%)
Grade 1	14 (41.2%)
Grade 2	11 (32.3%)
Grade 3	9 (26.5%)
**Lobular inflammation**	
None	1 (2.9%)
2 per foci	5 (14.7%)
2–4 foci	19 (55.9%)
>4 foci	9 (26.5%)
**Ballooning degeneration**	
None	3 (8.8%)
Few ballooned cells	20 (58.8%)
Many ballooned cells	11 (32.3%)
**NAS**	
<3	2 (5.9%)
3–4	11 (32.3%)
5–8	21 (61.8%)
**Portal inflammation (grade**)	
0	8 (24%)
1	11 (32%)
2	11 (32%)
3	3 (9%)
4	1 (3%)
**Fibrosis (stage**)	
0	4 (11.7%)
1a	8 (23.5%)
1b	5 (14.7%)
1c	1 (3%)
2	11 (32.3%)
3	4 (11.7%)
4	1 (3%)
**Enzyme levels, mean (range**)	
AST	55 IU/L (19–139)
ALT	91 IU/L (18–330)
GGT	109 IU/L (16–841)
ALP	105 IU/L (43–256)

ALP = alkaline phosphatase, ALT = alanine aminotransferase, AST = aspartate aminotransferase, GGT = gamma-glutamyl transferase, MASLD = metabolic dysfunction-associated steatotic liver disease, NAS = NAFLD activity score, SD = standard deviation.

### 3.3. Immunohistochemical analysis

Portal and central areas were visualized with the guidance of CD34, and GS slides evaluated comparatively in addition to H&E and MT slides. CD34 immunoreactivity was detected in hepatic and portal venules, as well as in periportal sinusoids in some cases.

An abnormal distribution of portal tracts, such as uneven proximity to each other and the central vein, was detected in 30 cases (88%) using CD34 and GS staining. The usual perivenular GS staining pattern was observed in 29 cases (85.2%). Weak perivenular or enlarged perivenular hepatocellular GS staining patterns, covering zones 3 and 2, were observed in 5 cases (14.7%). Uneven proximity of portal tracts was not observed in the control group (*P* < .001).

The mean percentage of portal tracts lacking bile ducts was significantly higher in the study group compared to the control group (*P* < .001). In the study group, the mean percentage of portal tracts with interlobular bile ducts was 61.32% ± 16.32%, whereas it was 93.6% ± 10.44% in the control group. The percentages of portal tracts without interlobular bile ducts were distributed as follows: <10% in one case, 10% to 49% in 25 cases, 50% to 75% in 7 cases, and >75% in one case. Applying the threshold of 50% for the diagnosis of bile duct loss, a total of 8 cases (24%) had “ductopenia.”

DR was observed in 25 cases (73.5%) with CK7 and CK19 immunohistochemistry. In the study group, 14 cases (41.2%) exhibited mild, 7 cases (20.6%) moderate, and 4 cases (11.8%) severe DR. In cases with weak perivenular or enlarged perivenular hepatocellular staining patterns with GS (5 cases), grade 3 steatosis and moderate to severe DR were observed (*P = .005*). No DR was observed in the control group.

Focal HPCs were detected in 5 (50%) cases, with a mean number of 2 ± 2 SD (range 0–6) in the control group. Given that the maximum number of HPCs observed in the control group was 6, this value was established as the threshold for determining the presence or absence of HPCs in the study group. Consequently, HPC activation was detected in 27 (79%) cases in the study group.

Biliary metaplasia was observed in 6 cases (18%), and all these cases showed stage 2 or 3 fibrosis.

**Figure 1.**
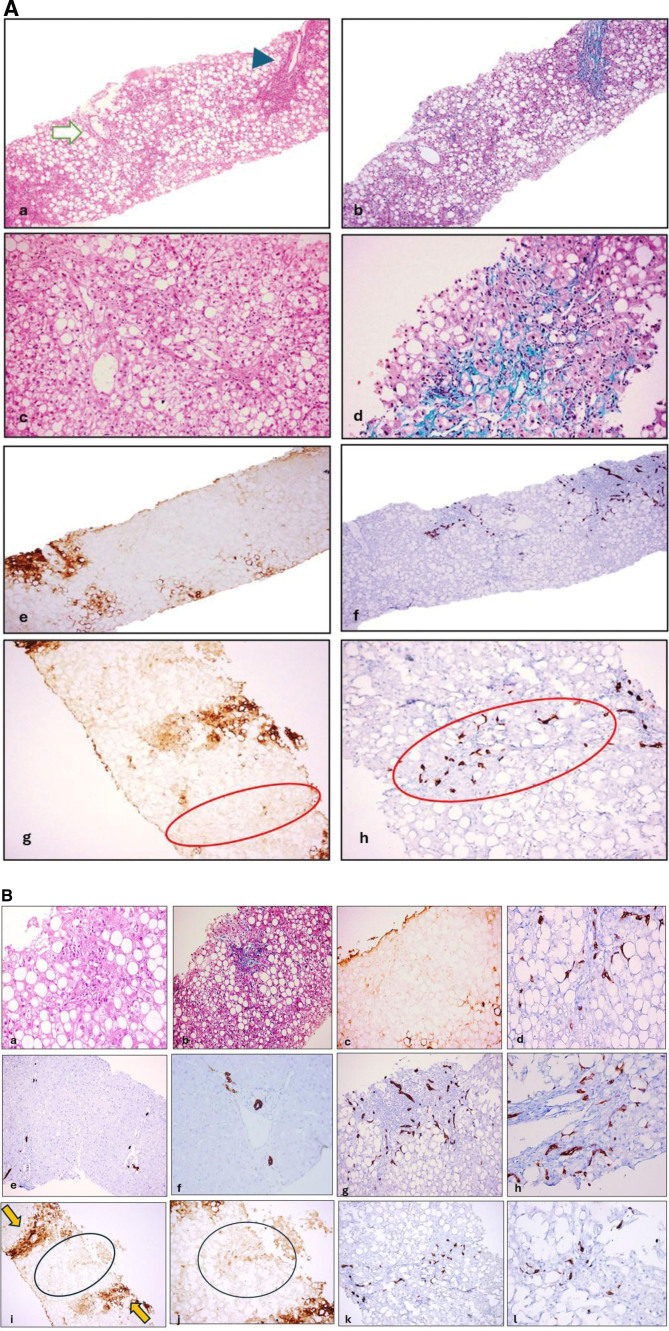
Histological and immunohistochemical (K7, K19, and GS) findings in MASH are shown. (A) An irregular distribution of macrovesicular steatosis in liver biopsy section stained with H&E. The central area (arrow) and the portal area (arrowhead) are marked (A). Portal fibrosis is observed with Masson-trichrome staining in the same area shown in a (B). At higher magnification, macrovesicular steatosis, ballooning degeneration, and lobular inflammation are noted in the pericentral area (c). The characteristic perisinusoidal fibrosis pattern is seen in the centrilobular area (d). With the guidance of GS (e and g) (positively stained hepatocytes around the hepatic veins), HPC activation and DR are highlighted with K7 staining at the periportal areas (f and h). Additionally, the absence of the interlobular bile duct in the portal tract is shown (h). The circled areas are the same areas in figures (g) (GS stain) and (h) (K7 stain). (B) An inflammatory focus is visible with H&E staining (A), while irregular fibrosis is observed in the same area with MT staining (B). The absence of GS expression confirms the portal tract location (c). HPC activation in periportal areas is demonstrated by K19 staining (d). In a control liver biopsy, normal K19 staining indicates the absence of HPC activation and/or DR (e, f). However, HPC activation and DR are observed in steatohepatitis (g, h). GS staining in zone 3 hepatocytes is indicated by an arrow (i), while the absence of GS staining in the circled area confirms the portal tract location (j). K19 staining in the same area shown in figure “j” highlights HPC activation, and the lack of K19 expression in this portal tract is consistent with bile duct loss (k). Another portal tract in a different section also shows the absence of a bile duct (l). DR = Ductular reaction, GS = glutamine synthetase, H&E = hematoxylin-eosin, K7 = cytokeratin 7, K19 = cytokeratin 19, MASH = Metabolic dysfunction-associated steatohepatitis, MT = masson-trichrome.

Histologic and K7, K19, and GS immunohistochemistry findings in MASH are shown in Fig. 1A and B.

### 3.4. Correlation between the histologic, immunohistochemical and laboratory findings

The degree of lobular inflammation was significantly correlated to the ballooning degeneration, the degree of portal inflammation, DR grade and the degree of fibrosis. As the score of lobular inflammation increased, the ballooning degeneration (*P* = .023), the portal inflammation score (*P* = .003), DR grade (*P* = .027), and degree of fibrosis increased (*P* = .003).

A significant correlation observed between the grade of steatosis and the grade of DR (*P = .03*). The ballooning degeneration was related to the degree of fibrosis (*P = .004*). No fibrosis was observed in 3 cases without ballooning degeneration. However, varying degrees of fibrosis were observed in 95% of the cases with few ballooned cells, and in all cases with many ballooned cells.

NAS and degree of fibrosis were significantly related to each other as well (*P* = .008). While no fibrosis was observed in 2 cases with NAS of < 3, varying degrees of fibrosis were detected in cases with NAS of 3 to 4 and 5 to 8. The portal inflammation was also found to have a significant relationship with both DR grade (*P* < .001) and the degree of fibrosis (*P* = .002).

**Figure 2.**
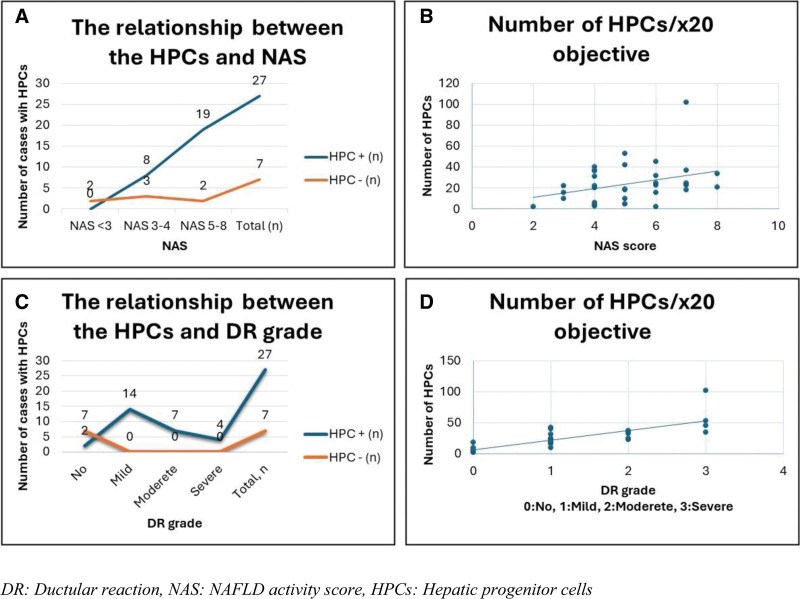
The correlation between HPCs, NAS, and DR grade. The presence of HPCs was found to be related to NAS (A), and the number of HPCs increasing with increase in NAS (B). HPCs were correlated with the DR grade. The greater the number of HPCs, the higher the grade of DR observed (C, D). DR = Ductular reaction, HPCs = hepatic progenitor cells, NAS = NAFLD activity score.

The mean number of HPCs was higher in the study group (mean 24.3 ± 19.1 SD) than the control group (mean 2 ± 2 SD) (*P < .005*). The presence of HPCs was found to be related to NAS (*P = .005*). Seventy-three percent of cases with NAS of 3–4 and 91% of the cases with NAS of 5–8 exhibited HPCs while no HPCs were detected in the cases with a NAS of < 3. In cases of elevated NAS, an increase in the HPC count (NAS < 3: 6 ± 5.6 SD, NAS 3–4: 21.5 ± 13.4 SD, and NAS 5–8: 27.6 ± 21.6 SD) was noted. Nevertheless, this association was not statistically significant (r: 0.33, *P* = .27). There was no significant relationship between the presence or number of HPCs and ballooning degeneration (*P* = .49, *P* = .26 respectively). The HPCs were correlated with the DR grade. The greater the number of HPCs, the higher the grade of DR observed (*P* = .002, *r*: 0.78). The relationship between HPCs, NAS and DR grades is shown in Fig. 2.

**Figure 3.**
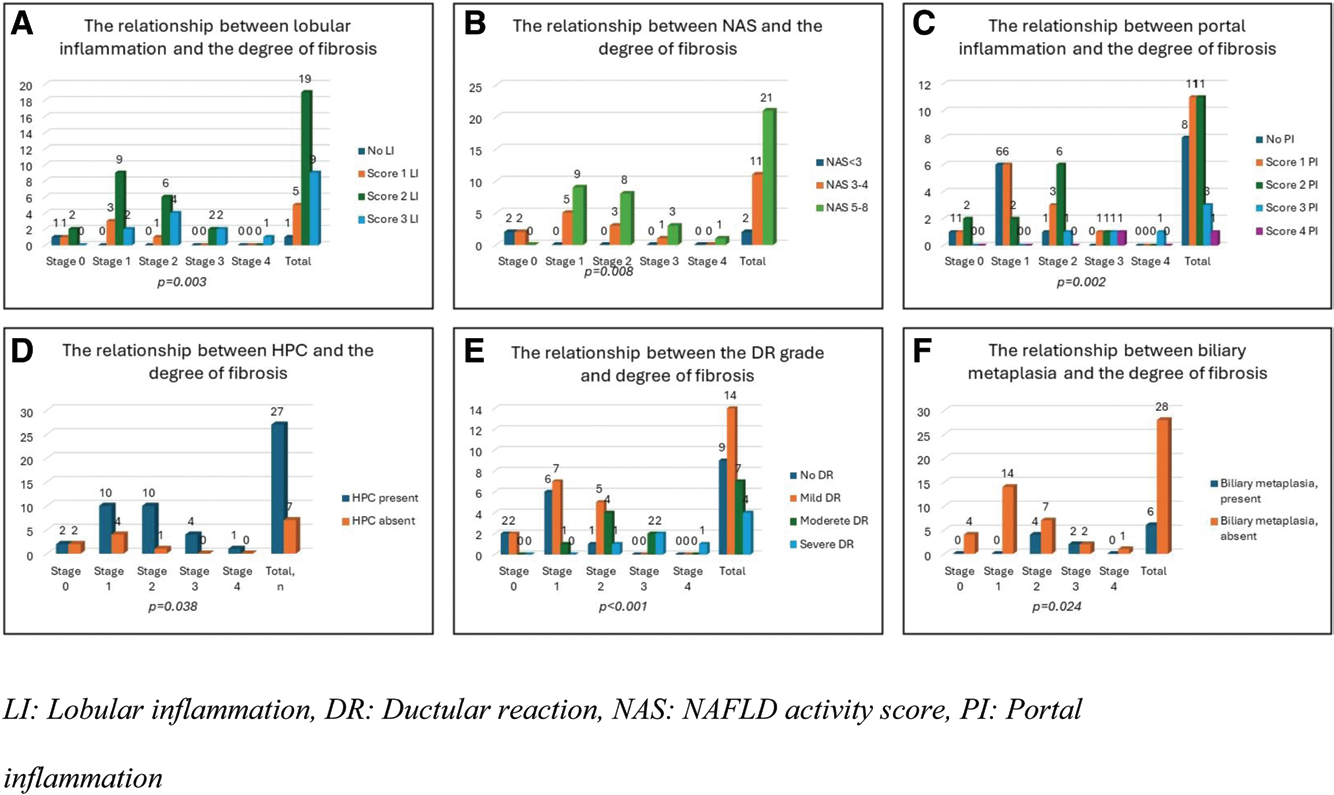
Parameters related to the degree of fibrosis. Lobular and portal inflammation and an increase in the NAS score, were associated with the stage of fibrosis (A–C). The presence of HPCs, biliary metaplasia, and DR grade were also related to the stage (D–F). DR = Ductular reaction, HPCs = hepatic progenitor cells, NAS = NAFLD activity score.

The HPCs were correlated with the degree of fibrosis (*P = .038*). No HPC was detected in 50% of stage 0 cases and 29% of stage 1 cases. In contrast, they were present in all cases classified as stage 2, 3, and 4. DR (*P* < .001) and biliary metaplasia (*P* = .024) were also significantly correlated with the degree of fibrosis. In stage 0 cases, no or mild DR was observed. However, with the progression of fibrosis, the severity of DR increased. Both stage 3 and stage 4 exhibited moderate to severe DR. Biliary metaplasia was observed in only 6 cases, all of which were at stages 2 and 3. The parameters related to degree of fibrosis are shown in Fig. 3.

No evidence of cholangitis or cholestasis was observed in any of the cases. Ductopenia was present in 24% (n:8) of the cases. No relationship was observed between ductopenia and NAS, portal inflammation, HPCs, DR, the degree of fibrosis, or elevated liver enzymes and hyperbilirubinemia.

Elevation in AST levels was correlated with lobular inflammation (*P* = .006), portal inflammation (*P* = .047), as well as elevation in ALT and ALP levels (*P* < .001, *P* = .017 respectively). All patients with elevated AST levels had lobular inflammation scores of 2 or 3.

Elevation in ALP was correlated with lobular inflammation (*P* = .006), portal inflammation (*P* = .019), and biliary metaplasia (*P* = .03). All patients with elevated ALP levels had lobular inflammation scores of 2 or 3. The ALP level was elevated in 4 out of 6 (67%) cases with biliary metaplasia.

No association was found between HPCs, DR, or bile duct loss and elevated levels of GGT or hyperbilirubinemia.

### 3.5. Multivariate analysis for the degree of fibrosis

The parameters (lobular inflammation, ballooning degeneration, portal inflammation, NAS, biliary metaplasia, HPCs, DR) found to be significantly correlated to the degree of fibrosis in the univariate analyses were evaluated in the multivariate analysis. Biliary metaplasia (OR: 2.157, 95% CI: [0.164–1.373]; *P* = .015) and DR (OR: 2.453, 95% CI: [0.355–1.439]; *P* = .02) were found to be independent factors related to the degree of fibrosis.

## 4. Discussion

We evaluated the histomorphologic findings in the liver biopsies of the patients clinically diagnosed as MASLD; and we analyzed the findings that may play a key role in the fibrogenesis and potentially may contribute to the disease progression.

In MASLD patients, the diagnosis of steatohepatitis is indicated by the presence of lobular inflammation and ballooning degeneration. We observed higher numbers of HPCs in the cases with higher NAS scores whereas no HPC activation was observed in the cases with lower activity scores. Studies have shown that HPC activation occurs due to the inhibition of the regenerative capacity of mature hepatocytes caused by oxidative stress, which is the key event in the development of steatohepatitis along with other liver diseases, in both animal models and humans.^[17]^

The unbalanced proximity of the portal tracts to each other and the central vein was observed in 88% of our cases through the evaluation of GS and K7 immunohistochemistries. This can be explained by the phenomenon known as parenchymal extinction, characterized by focal loss of hepatocytes, destruction of sinusoids, and obstruction of the small portal and hepatic veins resulting from conditions such as MASH or other liver diseases.^[27]^ An important feature of parenchymal extinction is that local vascular impairment delays regeneration. Consequently, tissue replacement from progenitor cells becomes the dominant pathway for repopulating injured regions.^[27,28]^ Our results highlight that recognizing the proximity of portal tracts to each other and to the central vein is important to avoid misinterpreting periportal HPCs as pericentral or zone 2-located, especially in cases where the interlobular bile ducts are not discernible within the portal tracts. This can be avoided by the addition of GS and K7/K19 immunohistochemistries in the routine pathological examination of the liver biopsies of MASLD patients.

In our study, both HPC and DR were significantly correlated with the degree of fibrosis even though the former lost its significance in the multivariate analyses. The increase in HPC number correlated with the increase in DR grade, indicating that progenitor cell activity might play an initiating role in DR development followed by liver fibrosis.^[29]^ This finding is consistent with previous studies.^[15,16,19,30]^

In multivariate analysis, DR was one of the parameters independently associated with the degree of fibrosis. An increasing DR retained an independent association with the stage of fibrosis, while the association with HPCs lost its statistical significance, as mentioned in a previous study.^[16]^ The loss of the impact of HPCs on fibrosis can be explained by the phenomenon where, in instances where 2 parameters demonstrate significant statistical dependence, one may lose its significance as an independent prognostic indicator. The interdependence between DR and HPCs was supported through multivariate analysis.

The relation between fibrosis, HPC, and DR can be explained by the impaired regeneration capacity of mature hepatocytes in MASH leading HPC activation followed by DR formation and fibrosis.^[15,29]^

We observed ductopenia in 8 cases (24%) as a curious finding and no relationship was found between ductopenia and NAS, portal inflammation, HPCs, DR, the degree of fibrosis, or elevated liver enzymes and hyperbilirubinemia. Since clinical and laboratory data are crucial when evaluating medical liver biopsies, it is important to emphasize that the presence of portal tracts lacking bile ducts and the presence of DR should not automatically prompt the pathologist to conclude accompanying biliary pathologies without elevated cholestatic liver enzyme levels. The clinical significance of ductopenia in such a cohort remains to be explained by further studies in larger series.

## 5. Conclusion

Our results showed that HPC and DR were closely associated with disease activity and degree of fibrosis and might be good indicators of disease progression in MASLD. As pathologists, we might integrate the degree of HPCs and the grade of DR in our pathology reports as these findings might contribute to the disease progression risk categorization of the patients.

## Author contributions

**Data curation:** Melek Büyük, Bilger Çavuş.

**Formal analysis:** Melek Büyük, Sidar Bağbudar, Bilger Çavuş, Mine Güllüoğlu.

**Investigation:** Melek Büyük, Neslihan Berker, Sidar Bağbudar, Bilger Çavuş, Mine Güllüoğlu.

**Methodology:** Melek Büyük, Neslihan Berker, Sidar Bağbudar, Mine Güllüoğlu.

**Supervision:** Mine Güllüoğlu.

**Writing – original draft:** Melek Büyük.

**Writing – review & editing:** Melek Büyük, Mine Güllüoğlu.
